# Do Medical Universities Students Use Cognitive Enhancers while Learning?—Conclusions from the Study in Poland

**DOI:** 10.3390/life13030820

**Published:** 2023-03-17

**Authors:** Anna Merwid-Ląd, Michał Passon, Paweł Drymluch, Maciej Głuszyński, Adam Szeląg, Agnieszka Matuszewska

**Affiliations:** 1 Department of Pharmacology, Wroclaw Medical University, J. Mikulicza-Radeckiego 2, 50-345 Wroclaw, Poland; 2 Faculty of Medicine, Wroclaw Medical University, J. Mikulicza-Radeckiego 5, 50-345 Wroclaw, Poland

**Keywords:** cognitive enhancers, Medical University students, online survey, dietary supplements, caffeine, nicotine, ginseng, ginkgo, theanine, lecithin

## Abstract

Background: Stress and everyday problems may impact memory and cognition. Therefore, many people use cognitive enhancers (CEs), sold for prescription, as over-the-counter drugs, or dietary supplements, believing they may help with everyday functioning. Our study was designed to answer whether taking CEs is common among Medical University students and to identify which substances are mainly used. Methods and Results: An anonymous online questionnaire was answered by 479 students of Medical (88%) and Dentistry (12%) Faculties in Poland. Women constituted the majority of respondents (63%). CEs were used by 53% of respondents, with the most frequent being caffeine, ginseng, nicotine, theanine, ginkgo, and lecithin. Some persons used CEs that are available only with a prescription. The most important reasons for the use of CEs were to increase arousal and improve concentration (mentioned by 81% and 73%, respectively). Over 65% of students experienced some undesired/adverse effects after taking CEs, with tachycardia being the most common, followed by sleep disturbances (reported by 51% and 40%, respectively). Conclusions: More than half of the respondents from the Medical and Dentistry Faculties reported using CEs, despite their unproven efficacy and not-well-established safety. This raises significant concern about the knowledge of young persons regarding CEs and should encourage universities to undertake educational actions.

## 1. Introduction

Cognitive enhancers (CEs) or neuroenhancers is the term given for substances or interventions that improve mental functioning in humans but are not necessary to restore or maintain good health. They may be pharmacological (e.g., drugs, supplements, nutraceuticals, and functional foods) and non-pharmacological (various brain stimulation methods) [1,2]. Many pharmacological cognitive enhancers do not have established efficacy, and only a few are registered for this purpose and are often used “off-label” [2]. In a rigorous meaning, “pharmacological cognitive enhancement” refers to the illegal consumption of substances or prescribed drugs by healthy individuals to increase concentration, memory, alertness, attention, and sometimes mood. Caffeine, energy beverages, or some herbal formulations such as ginkgo do not fit this definition, and they are called “soft neuroenhancers” [1]. Generally, using any pharmacological substance with unproven efficacy in healthy individuals, including students, raises many ethical concerns among healthcare professionals [2,3,4].

The use of CEs by students is a growing trend, and, e.g., the availability of nootropics via the Internet has increased dramatically during the last decades, with more than 700 novel psychoactive substances, including CEs, having appeared on the European market, the majority in the previous decade [5]. Many CEs are sold as dietary supplements (DS). Some of these compounds are newly synthesized, and others (especially of natural origin) have been known for ages and are used in traditional medicine worldwide. Many factors influenced the incredible popularity of DS. Among others, natural or herbal DS are considered safer than drugs and even sometimes more effective. The other important reason for the popularity of DS is that they are widely advertised in media, are readily available, and do not require medical consultations [6,7]. Many people do not understand the difference between drugs and DS correctly and use DS to treat various disorders or take them with drugs, resulting in harmful interactions [7]. The study performed in Poland in 2014 revealed that about 25% of questioned people incorrectly defined dietary supplements, and 41% of respondents claimed that DS might treat diseases [8].

Medical students must memorize much theory and learn many practical aspects during their studies, in most cases, concisely. It requires, besides individual predispositions, hard work and a variety of skills, and may generate frustration and stress. Stress intensity may be one of the factors influencing cognition [9]. It can lead to the use of compounds to improve memory or concentration, or to decrease tiredness and the need for sleep. Some authors found a significant relationship between the use of CEs and stress levels. Different studies have shown that in the pre-exam period, the consumption of CEs increased, as well as the intake of caffeine, nicotine, and alcohol [10,11]. As we also reported in our other study [12], Medical and Dentistry students estimated their stress levels as high (67.5% and 75.5%, respectively).

Additionally, increased cognitive capacity may lead to better results during the studies, a better position in the labor market after the studies, higher financial income, and overall well-being [13]. Medical students are a particular group that will advise patients in the future. Therefore, their attitude and acceptance of the use of CEs are essential [14]. Moreover, they may have easy access to prescription-only medicines used off-label as CEs (often from family members). What is essential, the decision about the use of CEs is not always an individual, autonomous choice, but might be a part of the person’s social life [15] or the effect of tremendous pressure on young persons to obtain almost perfect results [16].

Since there are many contradictory papers published about the prevalence of the use of CEs in different regions worldwide [11,17,18,19], we decided to evaluate this problem among medical students in Poland, primarily due to very scant data from Poland available in this field. Therefore, our study aimed to assess the frequency of the use of various substances with a potential impact on cognitive function among medical and dentistry students in Poland during the COVID-19 pandemic, the type of these compounds, and their effects on the questioned persons. Unlike students at other universities and faculties, medical students should have a greater awareness of the impact of these agents.

## 2. Materials and Methods

### 2.1. The Study Design and the Sample Size Calculation

The survey was created and prepared by the authors of the research. Ready-made online questionnaires or earlier published forms were not used. The questionnaire was pre-tested on a group of a few students at Wroclaw Medical University (excluded from further analyzes), which minimized the risk of questions’ ambiguity. After approval by the Bioethics Committee, the final version of a questionnaire was prepared using Google^®^ Forms. The survey link was shared with groups of Polish Medical and Dentistry Faculties students on the Facebook social network. It was also sent to the groups’ leaders at Wroclaw Medical University. Data to analyze were collected from the online anonymous and voluntary survey conducted between the 1 July 2021 and the 1 August 2021, and later, from the 3 November 2021 to the 2 January 2022.

The time of the data collection was during the pandemic; therefore, the online form was used due to the sanitary regime and epidemiologic situation. The sanitary recommendations at the Medical Universities changed very often, depending on the epidemiological state in Poland, e.g., from complete online learning at Wroclaw Medical University from March 2020 to January 2021, to hybrid learning with online tests and exams until September 2021. Additionally, restrictions were introduced regarding transferring documents in paper form with the preference for electronic records, when only possible. The written informed consent of respondents was not collected. Still, all participants were informed before the survey started that the participation was voluntary and anonymous. They were advised that they were able to quit the online survey form at any time without saving the answers.

The minimum sample size was calculated using the modified Cochran’s formula for sample size calculation in smaller populations.

Cochran’s formula for the infinitive population:

$$N_{0}=\frac{Z^{2}pq}{e^{2}}$$

Adjusted sample size:

$$N=\frac{N_{0}}{1+\frac{N_{0}-1}{population}}$$

where: *N*_0_ = sample size for infinite population; *Z* = *Z*-score (depends on the confidence level); *e* = the desired level of precision (margin of error); *p* = the proportion of the population (which has the attribute questioned); *q* = 1 − *p*; *N*—sample size adjusted for population.

The population proportion (*p*) describes a percentage of the value associated with the survey. If the value is not known, it should be assessed for 50% (the value which is the worst-case scenario percentage) as we have chosen for the calculations [20,21]. It is later expressed as 0.5 and *q* = 1 − 0.5, the margin of error is set for 5% (0.05), and the confidence level is 95%, which indicates that the *Z*-score is 1.96. Calculating the sample size *N*_0_ is 385 students. Adjusting to the population of about 42,000 medical and dentistry students in Poland, a minimum sample size of 381 students (men and women, Medical and Dentistry) are required for the study. The calculations were performed using the online Sample Size Calculator [20].

### 2.2. Ethical Approval

The study was conducted according to the Declaration of Helsinki guidelines and was approved by the Wroclaw Medical University Bioethics Committee (agreements KB-544/2021 from 22 June 2021 and KB-799 from 8 October 2021).

### 2.3. The Questionnaire Organization

The 12 questions in the survey were divided into four sections. The first section was a short description of the study, an introduction of the survey authors, information about the voluntary and anonymous character of the study, and the possibility of quitting the survey. The term “cognitive enhancers” was also explained in this part. The following section (questions 1–3) was about the standard demographic data, such as gender, age, and faculty (Medical or Dentistry). In Section 3, there was only one question about using cognitive enhancers (YES/NO). If the answer was “NO”, the survey was finished. If the answer was “YES”, the respondents filled the fourth part with questions about CEs. The questions from 5 to 11 were about the type of the CEs used, the main reasons for and frequency of the use of CEs, as well as the primary source of knowledge about them, the most significant pharmacological effects observed after the use of this type of supplements/drugs, and questions about the presence and nature of the adverse effects, noticed during the use of the CEs. The last question in this section was whether the COVID-19 pandemic increased the use of CEs in a group of Medical and Dentistry students.

English-language version of the survey consists of Attachment S1 in Supplementary Materials.

### 2.4. Statistical Analysis

TIBCO STATISTICA 13.3 PL Software (StatSoft, Kraków, Poland) was used for statistical analyses. The differences in categorical variables in the subgroups of students were compared by Pearson’s chi-square test.

The differences in the number of taken CEs were checked with the Mann–Whitney U test (after excluding the normality with the Shapiro–Wilk test). Additionally, logistic regression was performed to evaluate the impact of the analyzed independent features. As statistically significant was considered a *p*-value less than 0.05.

## 3. Results

### 3.1. Demographic Data

**Figure 1:**
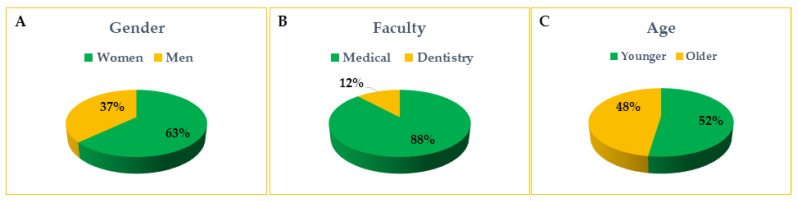
The primary demographic data of the respondents. Gender (**A**), Faculty (**B**), and age (**C**); Younger refers to students from 18 to 21 years of age, and Older refers to students over 21 years of age.

The primary demographic data of the participants are presented in Figure 1A–C. Four hundred and seventy-nine Medical and Dentistry Faculties students from Medical Universities in Poland participated in the survey. Women constituted the majority of respondents (302 women and 177 men). Most of the respondents were students of the Medical Faculty, and Dentistry students represented 12% of respondents. Students under the age of 21 and students aged 21 and over responded in a similar proportion (250 and 229 persons, respectively). Overall, the mean age of our participants was 21.96 years (SD = 2.08).

### 3.2. The Use of Cognitive Enhancers

**Figure 2:**
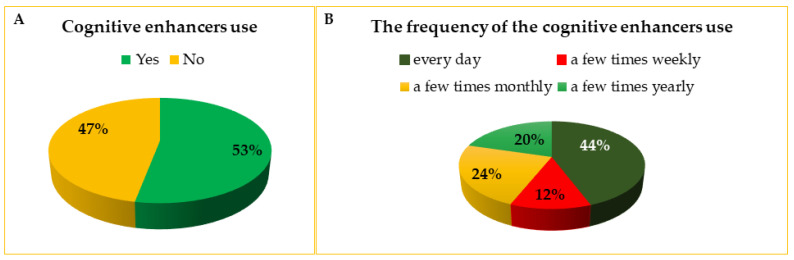
The use of cognitive enhancers by the students at Medical Universities in Poland (**A**) and the frequency of the cognitive enhancers use among respondents (**B**).

Of 479 participants, 255 students (53%) reported using cognitive enhancers (Figure 2A). After excluding the students taking caffeine as the only CE from this analysis, the prevalence of using at least one other cognitive enhancer was 41.9% (201 persons out of 479). In general, 44% of our respondents took the CEs every day. A similar number of students took CEs a few times per month and a few times per year (60 and 52 persons, respectively). The smallest group (11.8%) contained students reaching for CEs several times a week (Figure 2B).

**Figure 3:**
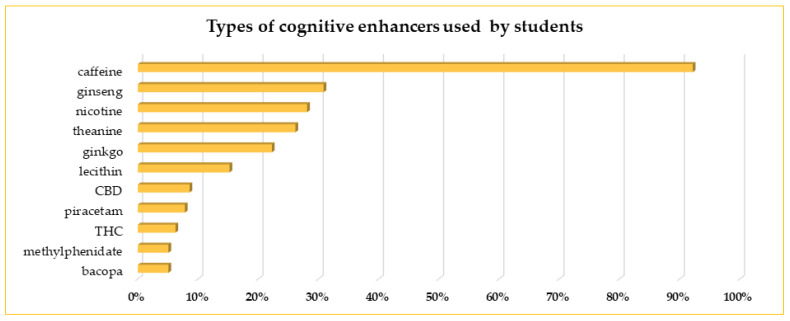
The most often used cognitive enhancers by Medical Universities students. In the figure are mentioned CEs used by at least 5% of the respondents; other CEs are mentioned in the text.

The most often chosen CE was caffeine, used by more than 90% of our respondents, followed by ginseng (31%). Respectively, 28%, 26%, and 22% of the students using CEs reported taking nicotine, theanine, and ginkgo. Few respondents choose lecithin (15%). Another cognitive enhancer mentioned by the users was cannabidiol (CBD). Not more than 10% of the students reported the use of prescription-only medicines. Twenty persons chose piracetam, and thirteen students chose methylphenidate from all students who declared using CEs. Sixteen students admitted to using tetrahydrocannabinol (THC), which is generally considered an illicit substance in Poland with few medical exemptions and is not registered as a CE (Figure 3). Not mentioned in Figure 3 were also modafinil (8 persons), vinpocetine (4 persons), memantine (3 persons), and nicergoline (2 persons). These substances were used by less than 3% of respondents.

**Figure 4:**
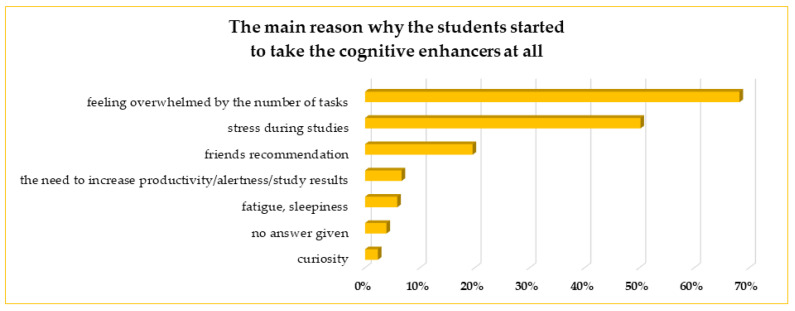
The main reasons why the students started the use of cognitive enhancers at all.

The most important reasons why the students started using cognitive enhancers are mentioned in Figure 4. The most often given answer was feeling overwhelmed with the number of tasks (68%), followed by stress during their studies (50%). Almost 20% of the respondents answered that the CEs were recommended by their friends, who suggested the efficacy of these compounds and that they may help the asking person cope with problems. Ten students did not give any answer for this question.

The overall use and frequency analysis were additionally performed in different subgroups of the respondents and presented in Table 1. No significant differences in the use of this type of dietary supplements were found between Dentistry and Medical students. In contrast, significantly more men than women used CEs (60.5% vs. 49%, respectively, *p* = 0.0154), and more often older (over 21 years of age) students than younger (up to 21 years of age) students took CEs (59.4% vs. 47.6%, *p* = 0.0098).

The results of logistic regression confirmed that men were more likely to take the CEs than women (*p* = 0.0170, OR: 1.59, CI: 1.09–2.33), and the students over 21 years of age than the younger students (*p* = 0.0199, OR: 1.55, CI: 1.07–2.23). No impact of the faculty on the use of CEs was noticed. Despite the slightly higher prevalence of the Dentistry students in taking the cognitive enhancers, the difference was insignificant (*p* = 0.2671, OR: 1.38, CI: 0.78–2.44).

The frequency of CEs usage in the studied subgroups of the respondents is presented in Table 2. Most respondents, independent of their sex, faculty, or age, took the CEs daily (from 40% among Dentistry students to 47.3% in the subgroup of female students). From 20.6% to almost 27% of students used the CEs a few times per week, with the lowest rate in the subgroup of older (above 21 years of age) respondents and the highest rate in the subgroup of the younger respondents. The lowest percentage of participants in the survey (8.1% of older students) used the CEs a few times per month, whereas a two times higher percentage of the younger persons took CEs with this frequency. No significant differences were found between the studied subgroups. However, the most pronounced differences (on the border of the statistical significance, *p* = 0.0522) were noticed between younger and older respondents considering taking CEs a few times per month or year.

The number of CEs the students took ranged from one preparation to as many as nine supplements. The mean value was 2.7 ± 1.5 (median value was 3, Q25 was 2, and Q75 was 4). No significant differences were found in the studied subgroups of respondents.

**Figure 5:**
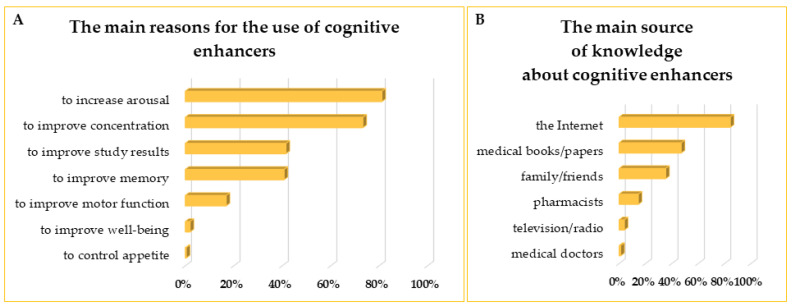
The main reasons for using cognitive enhancers among the respondents (**A**) and the primary sources of knowledge about cognitive enhancers for Medical Universities students (**B**).

Eighty-one percent of the students indicated the desire to increase general arousal as the main reason to take CEs, followed by the necessity to improve concentration indicated by 73% of the participants. Around ^2^/_5_ of the students wanted to enhance their study results or memory. The other, less frequent reasons to consume CEs are mentioned in Figure 5A. Over 80% of the respondents indicated the Internet as the primary source of knowledge about CEs. Only 1% of the students sought advice on CEs from medical doctors and about 15% from pharmacists. Quite often (47% of the respondents), medical books or papers were used as a source of information about CEs (Figure 5B).

**Figure 6:**
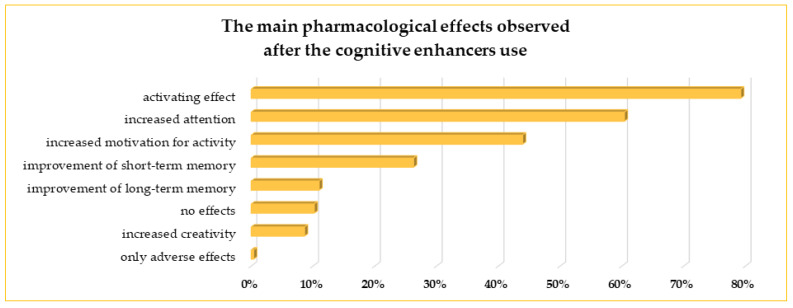
The main pharmacological effects noticed by the students during the use of cognitive enhancers.

Activating effect, increased attention, and increased motivation to undertake different activities were the most often mentioned as the main pharmacological effects during the use of CEs (79%, 60%, and 44% of respondents, respectively). Twenty-six and eleven percent of the students reported improved short-term and long-term memory, respectively. However, some students did not notice any positive pharmacological effects (10.2%), and one person indicated only adverse effects (Figure 6).

**Figure 7:**
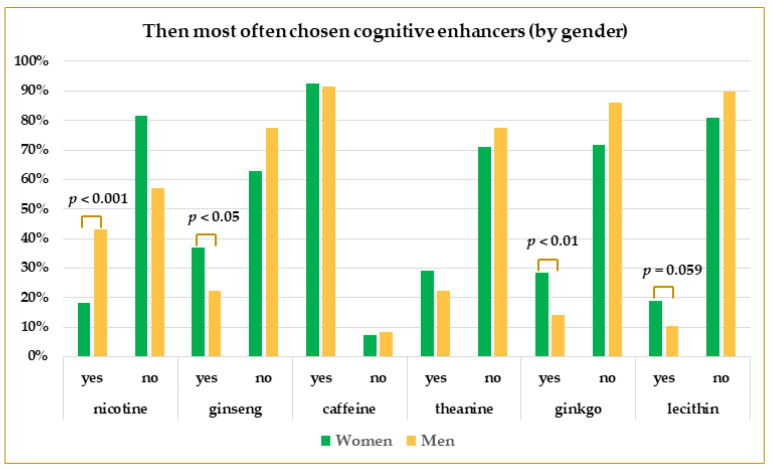
The prevalence of the use of main CEs by male and female students. *p*-values were calculated using Pearson’s Chi-square test.

Significantly more male students than females used nicotine (43% vs. 18%, *p* = 0.00002), whereas men were significantly less likely to choose CEs with ginseng (22% vs. 37%, *p* = 0.0121) and ginkgo (14% vs. 28%, *p* = 0.006). The use of caffeine and theanine was similar in both groups, and lecithin was slightly but insignificantly more often chosen by women than men (19% vs. 11%, *p* = 0.0586). The logistic regression confirmed a strong impact of gender on nicotine use (male vs. women, OR: 3.30, CI: (1.86–5.87), *p* = 0.0001). The detailed comparisons are presented in Figure 7 and Table 3.

**Figure 8:**
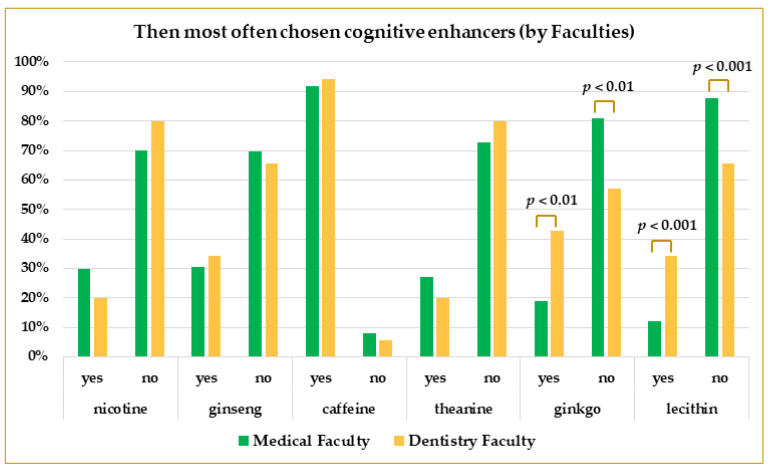
The prevalence of the use of main CEs by Medical and Dentistry students. *p*-values were calculated using Pearson’s Chi-square test.

Students of the Dentistry Faculty more often took ginkgo and lecithin than students of the Medical Faculty. Ginkgo was used by 43%, and lecithin by 34% of the dentistry students. At the same time, 19% of medical students took ginkgo-containing CEs, and 12% took lecithin-containing formulations. In both cases, the difference was significant (*p* = 0.0017 for ginkgo and *p* = 0.0008 for lecithin). The difference was confirmed by the results of the logistic regression analyses (OR: 2.69 for ginkgo and OR: 3.37 for lecithin, when comparing dentistry to medical students, both significant). The detailed comparisons are presented in Figure 8 and Table 3.

**Figure 9:**
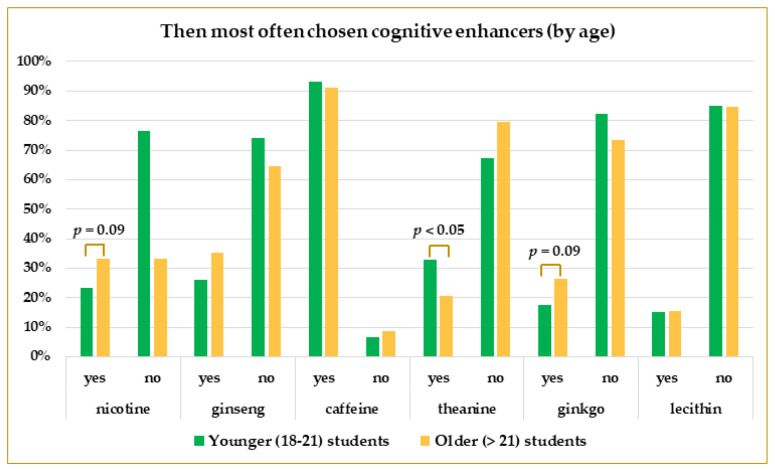
The prevalence of CEs use by younger (18–21 years of age) and older (over 21 years of age) students. *p*-values were calculated using Pearson’s Chi-square test.

As shown in Table 3, comparing the older students (over 21 years of age) with the younger participants, there was a significantly lower prevalence of taking theanine in the group of older students (OR: 0.54, CI: 0.31–0.95, *p* = 0.0335). Twenty-one percent of more senior students took theanine-containing CEs, whereas 33% of younger students took it (*p* = 0.0274). Some insignificant differences were noticed in the case of nicotine and ginkgo, which were slightly more often taken by older students. Almost the same percentage of younger and older participants used caffeine and lecithin (Figure 9).

### 3.3. The Adverse Effects of CEs

**Figure 10:**
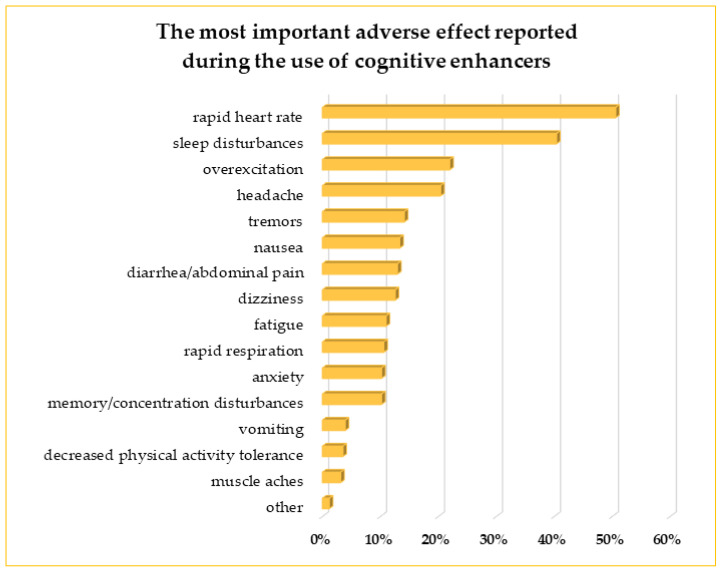
The most important adverse effects reported by respondents during the consumption of CEs.

Of 255 respondents who took CEs, 167 reported adverse effects, representing 65.5% of CEs users. More than 50% of students suffered from a rapid heart rate, and 40% complained about sleep problems. General overexcitation/agitation was reported by 22%, and headaches by 20% of CEs users. Other problems observed by the respondents were gastrointestinal disturbances with abdominal pain, nausea, and, less often, vomiting. Many students suffered muscle tremors, and eight had muscle aches. Also significant were dizziness, fatigue, anxiety, and problems with memory or concertation, reported by 10–13% of persons. Of note is the increased respiratory rate mentioned by 27 persons (11%). The frequency of the most troublesome adverse effects noted by the CEs users is presented in Figure 10.

### 3.4. The Impact of COVID-19 on the Use of CEs

One of the last questions was whether the COVID-19 pandemic and related to it distant learning increased the use of CEs. Most students (68.6%) who were CEs users answered that the COVID-19 pandemic did not increase the consumption of these supplements or drugs. However, 31.4% claimed they increased the use of CEs during the COVID pandemic. A similar ratio was found when the subgroups of female and male (31.8% and 68.2% vs. 30.8% and 69.2%), Medical and Dentistry (30.9% and 69.1% vs. 34.3% and 65.7%) or younger and older (31.9% and 68.1% vs. 30.9% and 69.1%) subgroups of students were analyzed.

## 4. Discussion

### 4.1. Demographic Data

According to the available data (as of 31 December 2021), the Universities in Poland educated about 1,218,200 students, and women constitute 58.4% of all studying persons [22]. Data from National Recovery Plan (NRP, published in June 2022) estimated a medical and dentistry population of 42,938 persons [23]. In our survey, women were in the majority (63%). It is similar to the proportion found in the general population of students and the data from the Wroclaw Medical University, where women constitute 72% of overall students and 62% and 66% in the subgroup of medical and dentistry students, respectively [24]. Similarly, in the cross-sectional study in France, published by Batisse et al. [25], dealing with the problem of using CEs, the respondents were mainly women (63.4%).

Among students in Poland, over 37,000 study at the Medical Faculty and almost 5000 at the Dentistry Faculty. Dentistry students constitute over 11.5% of this group [23]. Dentistry students in our survey were in the minority (12%). Still, the ratio of dentistry students to medical students represents the same tendency as in the population of Poland presented in NRP [23]. In this context, our respondents are a representative group because none of the subgroups (women and men, medical and dentistry students) were either over- or underestimated.

The mean age of the students in our study was 21.9 years. The ratio of younger (18–21 years of age) to older (over 21 years of age) students was 48% to 52%, which means that the students from both lower (preclinical) and higher (clinical) years of the studies were similarly willing to answer the questionnaire.

### 4.2. The Use of Cognitive Enhancers

Our survey revealed that 53% of medical and dentistry students used at least one substance considered as CEs during their studies. In other countries, the prevalence of CE use was lower, e.g., 32% in Portugal [18], and higher, e.g., 87.6% in Pakistan [10], than in our study. Even excluding from the consideration students using only caffeine, almost 42% of our respondents used at least one other CE. The prevalence was higher in men than women (60.5% vs. 49%) and in older than younger students (59.4% vs. 47.6%). In the study of the use of CEs among medical students in Lithuania, men also took CEs more often (almost three times greater prevalence) than women [19], and significant differences, with a higher percentage of men taking illicit or prescription drugs for cognitive enhancement, were also noticed in the study in Germany [26]. Similarly, in Iran, men were more likely to use CEs than women. An important factor influencing the decision was knowing someone who had used these kinds of drugs [27]. Ashraf Jahangeer et al. [10] noticed a similar tendency that men used the CEs more often than women, but differences were not so significant.

It is not easy to discuss the greater prevalence of CEs usage in older students in Poland. On the one hand, older students should be more aware of the potential harm of such substances, but on the other hand, they are more liberal about the use of CEs, what Erasmus and Kotze [14] noticed when asking the second and fifths-year medical students about the use of methylphenidate and the university policy regarding the prescription stimulants in non-medical purpose.

The leading source of knowledge about CEs cited by the students was the Internet, followed by medical books and friends or family members. As indicated by Nguyen et al. [28], the Internet was also the primary source of information about the safety of CEs, followed by the experience of peers, personal experience, and, in fourth place, scientific research. However, it is principal to mention that even though the cited earlier study pointed out the Internet was the most frequently used source of knowledge, it was, at the same time, indicated as the fourth most reliable source. The leading sources were scientific results and National Institute for Health and Care Excellence (NICE) guidelines. We did not ask if the Internet was considered a reliable or not source of knowledge, but it would be interesting to involve such questions in future questionnaires.

There were two leading reasons why the students started to take the CEs. The first was feeling overwhelmed by the number of different tasks during the studies, and the second was stress, mentioned by 68% and 50% of students, respectively. The third reason was the recommendation of friends who claimed that such substances might help to solve the students’ problems. Not too many papers directly describe the exact reason for starting CEs use, but Sümbül-Şekerci et al. [29] noticed that about 40% of pharmacological CEs users among medicine, pharmacy, and dentistry students stated to use CEs with the recommendation of their friends. In our study, fewer students (not exceeding 20%) started to use CEs after the advice of friends; however, friends or family members were the sources of knowledge about CEs for 35% of users.

Sharif et al. [30] found that students in the United Arab Emirates took the CEs mainly to increase academic performance, concentration, and alertness. Similar reasons were given by the Medical and Dentistry students in our survey, with the most common need to increase arousal, followed by improvement of concentration and study results. One other foremost reason almost 40% of participants mentioned was an improvement in memory, which was not a very important reason in the study of Sharif et al. [30]. However, in the recently published survey among medical students in Portugal [31], almost 44% of respondents used pharmacological CEs, and nearly 35% used easily available substances to increase memory. The most common reasons were similar to our study, to increase attention/focus and vigilance.

In the mentioned study by Sharif et al. [30], about one-third of students took the CEs daily. We noticed a higher prevalence of daily use, reaching about 44%, which might result from the everyday use of caffeine. In our study, about one-fourth of the respondents took the CEs a few times monthly, which may be consistent with the number of monthly tests to pass. Taking the CEs a few times per year may suggest using them mainly or exclusively before the final subjects’ exams, which are performed at most of the Medical Universities in Poland at the end of winter and summer semesters.

In our study, caffeine, in different forms, was the most often chosen CEs by our respondents (over 92%). It is estimated that about 80–85% of the adult world’s population uses caffeine because of caffeine-induced psycho-stimulation [31,32,33,34]. Our results are consistent with some other published data [33,35]. Ghalli et al. [35] reported using caffeine by almost 99% of students at the University in Dubai, and over 30% claimed to be addicted to caffeine.

Our students indicated that the main reasons to use CEs at all were to increase arousal and concentration, and the administration of caffeine may cause both these effects. Ágoston et al. [36] identified six main motivations for using caffeine and found some differences between subgroups of the Hungarian population. For example, younger participants (university students) had higher scores than older participants on “alertness”. Unfortunately, we did not ask the respondents about their motivation for choosing separate CEs. Of 200 respondents who indicated increased arousal as the reason for the use of CEs, 101 were younger, and 99 were older students. However, this is still the group of young users.

Ginseng was the second cognitive enhancer, just after caffeine, chosen by the students in our survey (more often by women than men). It was found that single doses of ginseng may improve the accuracy of memory tasks, working memory, or increased speed of attention task performance [37], and long-term use may positively impact cognitive functions in the future [38].

According to estimates, about one-third of the adult Polish population smoke, with 36.9% of men and 24.4% of women [39,40] and over 35% of young people (15–25 years of age) declaring themselves regular smokers [40]. In our study, 28% of the CEs users reported nicotine use in different forms, with significantly higher prevalence in men than women, which is consistent with the general trend in Poland. In contrast to the study of Zielińska-Danch, who described greater use of different tobacco products in the group of school-aged (15–19 years) participants than in the students’ group (19–25 years), in our study, the older students were more willing to use nicotine than the younger students. The cognitive enhancement properties of nicotine are still the area of various studies, but as was revised by Valentine and Sofuoglu [41], nicotine, in some range of doses, may improve cognitive functions. However, students also use nicotine for purposes other than typical cognitive enhancers. In some countries nicotine is used to cope with stressful situations and for recreation, esp. waterpipes [42].

A significant problem might be using prescription-only drugs, such as piracetam, modafinil, or methylphenidate, for cognitive enhancement. It was noticed that despite generating a variety of legal, ethical, and health concerns, there was a tremendous increase in the production of nootropics. Considering methylphenidate only, global production increased from a few tons in the last decade of the 20th century to more than seventy tons in the second decade of the 21st century [43].

From these three controversial drugs, at this moment in Poland, piracetam is available on prescription, methylphenidate—is on prescription but as a controlled drug, and modafinil is available on medical prescription for restricted use. According to the available Summaries of Product Characteristics (SmPC) [44], modafinil is registered in Poland in the treatment of excessive sleepiness associated with narcolepsy, and methylphenidate is part of a comprehensive treatment program for attention deficit hyperactivity disorder (ADHD). According to the SmPC, piracetam has the broadest indications including treatment of myoclonus of cortical origin, central and peripheral dizziness, or treatment of dyslexic disorders in children (simultaneously with speech therapy). Clinically is also used managing of cognitive disorders in dementia syndromes, except for Alzheimer’s disease.

It is disconcerting that as many as 7.8% of students used piracetam, 5.1% methylphenidate, and 3.1% modafinil as CEs when we compare it, e.g., with the data from Italy [33] when only 0.6% of respondents used the prescription-only medications over the last month. On the other hand, in the group of Lithuanian medical students [19], the prevalence of nootropics (piracetam or vinpocetine) use was 4%, but it is still less than in our survey. Among different pharmacological CEs, modafinil was the most often chosen substance by the students in the study from the United Kingdom [28], and methylphenidate was more than 20 times less frequently. Depending on the availability of such drugs in different countries, the prevalence of psychostimulant medicines varies greatly [33]. Whereas the use of modafinil or methylphenidate was often the main or additional aim of the studies and also described in reviews [45,46], the use of piracetam, which in Poland is relatively easy to buy, when compared with the two other drugs, is not often the purpose of the experiments.

We did not ask in the questionnaire where the students buy the CEs, esp. the prescription-only medicines. In Portugal [31], over 50% of medical students taking the pharmacological CEs were prescribed these drugs by general practitioners or psychiatrists despite a lack of medical indications for such medications. Additionally, students obtained prescription-only substances from colleagues, family members, or friends, and from the Internet. It indicates a problem with healthcare professionals’ inappropriate prescribing of pharmacological CEs by and their attitude toward CEs use. In the study of Ram et al. [3] in New Zealand, participants recruited from professionals (pharmacists, general practitioners, nurses, lawyers, and psychiatrists) strongly disagreed with the statement that “it was fair to allow university students to use CEs for cognitive enhancement, to concentrate, or to increase alertness/stay awake”, as well as strongly disagreed with the thesis that “it is ethical for students without a prescription to use cognitive enhancers for any reason”. It was not checked in this study, but it may be a noteworthy area of further research in Poland. Moreover, physicians and University teachers are a group with an increased risk of CEs use [47].

### 4.3. The Adverse Effects of CEs

The most common adverse effects mentioned by the students taking the CEs were rapid heart rate (over 50%) and sleep disturbances (40%). This observation is unsurprising because students (over 90%) often used caffeine as a CE. It was found that caffeine may produce rapid positive inotropic and chronotropic effects on the cardiovascular system and, in toxic doses, may cause life-threatening arrhythmias [34,48]. Caffeine can activate various brain areas [48], which is the reason for increased alertness but may lead to sleeping problems. It may induce tremors [34]. Considering different reported complaints from the gastrointestinal tract, we hypothesize that caffeine may also be one of the most important reasons due to its effect on gastric mucosa, potentiating gastric acid secretion and gastrointestinal motility [32,34]. However, Repantis et al. [49], in a controlled study of the effects of methylphenidate, modafinil, and caffeine (single dose of 200 mg) on cognitive enhancement, did not notice either increased heart rate or blood pressure during the whole study. In our study, we relied only on the symptoms reported by the respondents and not on objective measurement results. We did not ask about other factors (e.g., stress level), which may activate the sympathetic system and lead to tachycardia.

Because our respondents took very often more than one CE (over 72% took at least two different CEs), it is difficult to say which one was the most responsible for some reported adverse effects or if these adverse effects were not the result of interactions between CEs.

### 4.4. The Impact of COVID-19 on CEs Use

In our survey, over 31% of students reported the increased use of CEs during the COVID-19 pandemic, but without differences in the studied subpopulations of the respondents. The observations from Germany [50] from the time before and after the pandemic indicated that the prevalence of the use of CEs was similar in 2019 and 2020 and a little bit lower in 2021. Still. in general, it was about three times lower than found in our study. However, it is worth mentioning that we ask not only about psychostimulants or non-medical use of drugs but also about readily available dietary supplements. Our observations of the use of CEs during the COVID-19 time was similar to the overall tendency we found in our earlier study [12] about the use of dietary supplements for stress, anxiety, depression, or sleeping problems among student at Wroclaw Medical University. Most students did not change the pattern of DS use or took them less often, but 18% took this kind of DS more often, 19% started to take them during the pandemic and about 11% took more different kinds of DS. Although psychological distress is associated not only with COVID but also depends on other factors, overall, the COVID-19 pandemic negatively influenced the mental health of medical students [51], which subsequently may affect cognitive skills. It is impossible to avoid stress in everyday life, and pressure is necessary to create survival responses, but it may also affect cognitive functions and cause different behavioral disorders [52]. In our other survey among students at Wroclaw Medical University, over 70% of respondents declared a high or very high stress level [12], resulting in the use of different dietary supplements advertised as suitable for stress, anxiety, depression, or sleeping problems. It is well known that such factors as stress may impair cognitive function and negatively affect the student’s performance during the examination, as was found in a group of first-year medical students in India [53]. It might be one of the crucial reasons for taking CEs. We did not directly ask our respondents about the exact reasons for increased CEs during the COVID-19 pandemic, which may be interesting to study, especially in comparison to the current situation, when the Universities’ life has become almost routine.

## 5. Conclusions

The study revealed that many (53%) of the Medical and Dentistry students at Polish Medical Universities took different cognitive enhancers not only available without a prescription but also some substances registered as prescription-only medicines and even illegal once. Additionally, aside from some pharmacological effects, such as activation or increased attention, many of the CE users experienced significant adverse effects. The study revealed the scale of the problem and raises questions about how to improve medical students’ awareness of the risk associated with these types of preparations. The second, but no less important problem, is to consider how to minimize the desire and necessity to take CEs by students at medical universities by better organizing the study courses to optimize the use of study time by the students’ and by the reduction of unnecessary stress.

## Figures and Tables

**Table 1:** The general use of cognitive enhancers in various subgroups of respondents. *p*-values were calculated using Pearson’s Chi-square test.

The Overall Use of Cognitive Enhancers				
		YES	NO	*p*-Value
Gender	Women (*n* = 302)	49%	51%	*p* = 0.0154
	Men (*n* = 177)	60.5%	39.5%	
Faculty	Medical (*n* = 421)	52.3%	47.7%	*p* = 0.2471
	Dentistry (*n* = 58)	60.3%	39.7%	
Age	Students 18–21 years of age (*n* = 250)	47.6%	52.4%	*p* = 0.0098
	Students over 21 years of age older (*n* = 229)	59.4%	40.6%	

**Table 2:** The frequency of the cognitive enhancers’ usage in various subgroups of respondents. *p*-values were calculated using Pearson’s Chi-square test.

The Frequency of the Cognitive Enhancers’ Usage						
		Every Day	A Few Times per Week	A Few Times per Month	A Few Times per Year	*p*-Value
Gender	Women (*n* = 148)	47.3%	23%	12.2%	17.5%	*p* = 0.5355
	Men (*n* = 107)	40.2%	24.3%	11.2%	24.3%	
Faculty	Medical (*n* = 220)	45%	23.2%	11.3%	20.5%	*p* = 0.9244
	Dentistry (*n* = 36)	40%	25.7%	14.3%	20%	
Age	Students 18–21 years of age (*n* = 119)	42%	26.9%	16%	15.1%	*p* = 0.0522
	Students over 21 years of age (*n* = 136)	46.3%	20.6%	8.1%	25%	

**Table 3:** Impact of gender, faculty, and age on the intake of the most often used cognitive enhancers. Analyses were done using logistic regression (OR—odds ratio, CI—confidence interval).

	Dependent Factors	The Use of Nicotine	The Use of Ginseng	The Use of Caffeine	The Use of Theanine	The Use of Ginkgo	The Use of Lecithin
Independent Factors							
		OR	OR	OR	OR	OR	OR
		(95% CI)	(95% CI)	(95% CI)	(95% CI)	(95% CI)	(95% CI)
		B Coefficient	B Coefficient	B Coefficient	B Coefficient	B Coefficient	B Coefficient
		*p*-Value	*p*-Value	*p*-Value	*p*-Value	*p*-Value	*p*-Value
Gender (ref. Women)		3.30	0.48	0.91	0.67	0.45	0.57
		(1.86–5.87)	(0.27–0.85)	(0.36–2.31)	(0.38–1.22)	(0.23–0.88)	(0.27–1.23)
		1.19	−0.73	−0.09	−0.39	−0.80	−0.56
		*p* = 0.0001	*p* = 0.0124	*p* = 0.8449	*p* = 0.1939	*p* = 0.0193	*p* = 0.1525
Faculty (ref. Medical)		0.73	0.99	1.47	0.64	2.69	3.37
		(0.29–1.81)	(0.46–2.15)	(0.32–6.77)	(0.26–1.57)	(1.25–5.81)	(1.48–7/65)
		−0.32	−0.01	0.39	−0.45	0.99	1.21
		*p* = 0.4877	*p* = 0.9784	*p* = 0.6173	*p* = 0.3266	*p* = 0.0115	*p* = 0.0037
Age (ref. Students 18–21 years of age)		1.66	1.58	0.74	0.54	1.67	0.97
		(0.93–2.95)	(0.92–2.74)	(0.29–1.87)	(0.31–0.95)	(0.90–3.13)	(0.48–1.97)
		0.50	0.46	−0.31	−0.62	0.52	−0.03
		*p* = 0.0868	*p* = 0.1002	*p* = 0.5198	*p* = 0.0335	*p* = 0.1056	*p* = 0.9377

## Data Availability

Data are available on request.

## Supplementary Materials

The following supporting information can be downloaded at: https://www.mdpi.com/article/10.3390/life13030820/s1, the English-language version of the questionnaire is available as Supplementary Attachment S1.
