# Supplementary material for: Do Medical Universities Students Use Cognitive Enhancers while Learning?—Conclusions from the Study in Poland
Source: Life (Basel). 2023 Mar 17;13(3):820. doi: 10.3390/life13030820 (PMC10056889; doi:10.3390/life13030820)
Supplement: Supplementary file 1 [file life-13-00820-s001.zip › life-2219698-supplementary.pdf]

For the purposes of this survey, cognitive enhancers are substances you take to improve your mental abilities.

1. Faculty

- Medical
- Dentistry

2. Gender

- woman
- man

3. Age (write the year of your birth)

- ....

4. Have you ever used any substance to improve cognitive functions?

- YES
- NO

5. What kind of cognitive enhancer do you use? You can mark more than one answer.

- theanine
- nicotine
- caffeine
- *Bacopa monnieri*
- gingko
- ginseng
- lecithin
- CBD
- illicit substances (THC, cocaine, amphetamine)
- modafinil, armodafinil
- vinpocetine
- nicergoline
- piracetam, oxiracetam, aniracetam
- tolcapone
- levodopa
- rivastigmine, donepezil
- memantine
- methylphenidate
- desipramine
- atomoxetine
- other:

6. What made you decide to start taking cognitive enhancers? You can mark more than one answer.

- I felt overwhelmed by the number of tasks
- stress during studies
- friends recommended these substances to me, claiming that they could help me
- other:

7. How often do you take cognitive enhancers?

- every day
- a few times per week
- a few times per month
- a few times per year

8. For what purpose do you use cognitive enhancers? You can mark more than one answer.

- to improve attention
- to improve memory
- to have better results during studies
- to increase arousal
- to improve motor functions
- other:

9. What was your main source of knowledge about cognitive enhancers? You can mark more than one answer.

- the Internet
- radio and/or TV
- family and/or friends
- pharmacy
- medical books
- other:

10. What kind of pharmacological effects have you noticed during the use of cognitive enhancers? You can mark more than one answer.

- they improved my short-term memory
- they improved my long-term memory
- the activate me
- they increase my attention
- they increase my motivation to act
- they increase my creativity

11. What adverse effects did you notice during the use of cognitive enhancers?

- increased heart rate
- increased respiratory rate
- memory and concentration disturbances
- decreased tolerance of physical activity
- sleeping problems

- nausea
- vomiting
- diarrhea
- headaches
- overstimulation
- anxiety
- muscle aches
- tremors
- fatigue
- other:

12. Did the COVID-19 pandemic and remote learning increase the frequency of taking the cognitive enhancers by you?

- YES
- NO
